# Ticagrelor and clopidogrel suppress NF-κB signaling pathway to alleviate LPS-induced dysfunction in vein endothelial cells

**DOI:** 10.1186/s12872-019-01287-1

**Published:** 2019-12-30

**Authors:** Zhuyin Jia, Yiwei Huang, Xiaojun Ji, Jiaju Sun, Guosheng Fu

**Affiliations:** 1 Department of Cardiology, Sir Run Run Shaw Hospital, Zhejiang University School of Medicine, Hangzhou Zhejiang, China; 2 Department of Cardiology, Wenzhou Central Hospital, Wenzhou, Zhejiang China; 3 Key Laboratory of Cardiovascular Intervention and Regenerative Medicine of Zhejiang Province, Hangzhou Zhejiang, People’s Republic of China

**Keywords:** Ticagrelor, Clopidogrel, LPS-induced dysfunction, NF-ΚB signaling pathway, HUVECs

## Abstract

**Background:**

Ticagrelor and clopidogrel, P2Y_12_ receptor antagonists, can prevent thrombotic events and are used to treat cardiovascular diseases such as acute coronary syndrome and chronic obstructive pulmonary disease, in which inflammation is involved. Moreover, NF-B is the central regulator of inflammation. Thus, we suspected that ticagrelor and clopidogrel are involved in the regulation of the NF-ΚB signaling pathway.

**Methods:**

After human umbilical vein endothelial cells (HUVECs) were cultured with ticagrelor or clopidogrel and given lipopolysaccharide (LPS) and CD14, the mRNA levels of related inflammatory factors, the protein level and subcellular localization of molecules in the NF-ΚB signaling pathway, cell viability, apoptosis and the cell cycle, cell migration, and vascular formation were detected using quantitative polymerase chain reaction (qPCR), western blotting and immunofluorescence assay, CCK-8, flow cytometry, transwell assay, and matrigel, respectively. All data was expressed as the mean ± S.D. The statistical significance of data was assessed by an unpaired two-tailed t-test.

**Results:**

Ticagrelor and clopidogrel can inhibit the degradation of IKBα and phosphorylation of p65, prevent p65 from entering the nucleus, reduce the production of TNFα, IL-1, IL-8, IL-6 and IL-2, and alleviate the decrease in cell viability, cell migration and angiogenesis, the changes of cell cycle and apoptosis induced by LPS.

**Conclusions:**

Ticagrelor and clopidogrel alleviate cellular dysfunction through suppressing NF-ΚB signaling pathway.

## Background

Clinically, ticagrelor and clopidogrel, antiplatelet agglutination agents, are commonly used in combination with percutaneous coronary intervention (PCI) for acute coronary syndrome (ACS) [1]. They cure ACS by targeting the platelet P2Y_12_ adenosine diphosphate (ADP) receptor to inhibit platelet aggregation and reduce thrombosis, and the inhibitory effect of ticagrelor on the P2Y_12_ receptor is reversible, whereas inhibitory effect of clopidogrel is irreversible [2].

Some studies have indicated that inflammatory cytokines are involved in the initiation and progression of atherosclerosis which is one of the pathological features of ACS [3]. In addition to triggering thrombus formation at the site of atherosclerotic plaque rupture, platelets also release proinflammatory mediators and interact with other related cells, while antiplatelet therapy can reduce the levels of inflammatory cytokines [4]. Thus, inflammation plays an important role in ACS. Further research showed that NF-ΚB, a central regulator of inflammation, which is involved in various inflammatory diseases, is associated with susceptibility to ACS [4]. Furthermore, long-term administration of clopidogrel after severe coronary artery injury reduces inflammation via inhibition of NF-ΚB and activator protein 1 activation in pigs [1]. There are few reports on the mechanism of P2Y_12_ receptor antagonist-mediated inhibition of inflammation. Therefore, we wanted to know how P2Y_12_ receptor antagonist, including ticagrelor and clopidogrel, can regulate the NF-ΚB signaling pathway and reduce inflammation.

In the study, we detected the mRNA levels of related inflammatory factors, the protein level and subcellular localization of molecules in the NF-ΚB signaling pathway, cell viability, apoptosis, the cell cycle, cell migration, and vascular formation, after treating human umbilical vein endothelial cells (HUVECs)stimulated by lipopolysaccharide (LPS) and CD14 with ticagrelor or clopidogrel.

## Methods

### Preliminary experiment

#### Cell proliferation assay

HUVECs (FuHeng Cell Center, Shanghai, China, FH0278) were incubated with ticagrelor (0 μM, 5 μM, 10 μM, 20 μM, 50 μM, 100 μM) clopidogrel (0 μM, 5 μM, 10 μM, 20 μM, 50 μM, 100 μM), separately, for 12 h, 24 h or 48 h. Then cell viability was determined by CCK-8 (Biosharp, BS350B).

### Formal experiment

#### Cell culture and treatment

HUVECs were cultured in complete growth medium that was F12K containing 10% fetal bovine serum (FBS) and 1% Penicillin-Streptomycin Solution at 37 °C with 5% CO_2_.

HUVECs were treated with complete growth medium supplemented with DMSO (as control), ticagrelor, clopidogrel, DMSO plus LPS and CD14, ticagrelor plus LPS and CD14, or clopidogrel plus LPS and CD14, separately, for 16 h. The concentrations of these compounds are shown in the Table 1.

**Table 1:** The concentrations of these compounds

Compounds	Concentrations
Ticagrelor	20 μM
Clopidogrel	20 μM
LPS	10 ng/mL
CD14	1 μg/mL

#### Cell proliferation assay

Cells were seeded in 96 well culture plates (2000 cells/well). After the cells were incubated with the indicated compounds for 16 h. Finally, cell viability was tested with CCK8 reagent. We evaluated cell viability by measured the absorbance at 450 nm.

#### Western blot assay

Whole cell extracts were lysed in RIPA Lysis buffer (Beyotime, P0013B) containing 1 mM phenylmethylsulfonyl fluoride (PMSF). Then protein concentration of lysates was determined by BCA protein concentration determination kit (Beyotime, P0010). Cell lysates containing equal amount protein were resolved on a 10–12% sodium dodecyl sulfate polyacrylamide gel electrophoresis and then transferred to a PVDF membrane (Millipore, IPVH00010). After separate incubation with rabbit anti-p65 (CST, #8242), rabbit anti-p-p65 (CST, #3033), rabbit anti-MMP2 (proteintech, 10,373–2-AP), rabbit anti-MMP9 (proteintech, 10,375–2-AP), rabbit anti-E-cadherin (proteintech, 20,874–1-AP), rabbit anti-IKBα (abcam, Ab32518), rabbit anti-ICAM-1 (proteintech, 10,831–1-AP), rabbit anti-VCAM-1 (Affinity, DF6082), rabbit anti-E-selectin (proteintech, 20,894–1-AP), rabbit anti-GAPDH, mouse anti-P-selectin (proteintech, 60,322–1-Ig), mouse anti-MCP-1 (Affinity, BF0678), followed by horseradish peroxidase-conjugated secondary antibody, the membranes were visualized by ECL chemiluminescence.

#### RNA extraction and quantitative polymerase chain reaction (qPCR)

Total RNA was extracted using TRIzol reagent (Ambion, 15,596–026), and reverse transcription was accomplished with HiScript Reverse Transcriptase (VAZYME, R101–01/02). The reverse transcription products were amplified with SYBR Green Master Mix (VAZYME, Q111–02) according to the manufacturer’s instructions. The data were normalized according to the level of GAPDH expression in each individual sample. The qPCR primers are listed in Table 2.

**Table 2:** The qPCR primers

Name	Primer	Sequence
homo GAPDH	Forward	5′- TCAAGAAGGTGGTGAAGCAGG − 3′
	Reverse	5′- TCAAAGGTGGAGGAGTGGGT − 3’
Homo TNFα	Forward	5′-CCCATGTTGTAGCAAACCCTC − 3’
	Reverse	5′-AGAGGACCTGGGAGTAGATGA − 3’
	Reverse	5′-GTGCCTCTTTGCTGCTTTC − 3’
Homo IL-1	Forward	5′-CGAATCTCCGACCACCACTA − 3’
	Reverse	5′-AGCCTCGTTATCCCATGTGT − 3’
Homo IL-6	Forward	5′-GGTCCAGTTGCCTTCTCCC − 3’
	Reverse	5′-GTGCCTCTTTGCTGCTTTC − 3’
Homo IL-2	Forward	5′-CAACTCCTGTCTTGCATTGC-3’
	Reverse	5′-TGTGAGCATCCTGGTGAGTT − 3’
Homo IL-8	Forward	5′-GACATACTCCAAACCTTTCCACCCC-3’
	Reverse	5′-CAAAAACTTCTCCACAACCCTCTGC −3’

#### Immunofluorescence assay

The cells were incubated with the indicated compounds and then fixed for 15 min in 4% paraformaldehyde in 1 × phosphate-buffered saline (PBS) pH 7.4. The fixed cells were permeabilized for 20 min with 0.5% Triton X-100 in 1 × PBS and then blocked in 1× PBS with 1% bovine serum albumin for 30 min. The cells were incubated with the appropriate primary rabbit anti-p65 (CST, 8242S) and then stained with Alexa Fluor Cy3-labeled goat anti-rabbit immunoglobulin G (BOSTER, BA1032) and DAPI (Beyotime, C1002), separately. The subcellular localization of p65 was visualized using inverted fluorescence microscope (magnification, × 400).

#### Apoptosis assay

After incubated with the indicated compounds for 16 h, the cells were harvested and stained with APC/7-AAD apoptosis kit (SUNGENE BIOTECH, AO2001-11A-H), and then were analyzed by flow cytometry.

#### Cell cycle assay

After incubated with the indicated compounds for 16 h, the cells were harvested and stained with cell cycle kit (KeyGEN BioTECH, KGA512), and then were analyzed by flow cytometry.

#### Cell migration assay

After treated with the indicated compounds for 16 h, HUVECs were resuspended in serum-free F12K (2.5 × 105 cells/mL), 200 μL was added to the upper chambers, and complete growth medium was added to the lower chamber. After 24 h incubation, cells which migrated to the lower face of the membrane were fixed with 70% ethanol solution and stained by 0.5% crystal violet. After washed by PBS for 3 times, the migrating cells were observed under a microscope and photographed (magnification, × 200).

#### Matrigel assay

The cells were incubated with the indicated compounds for 16 h, and then cultured for 6 h in 24-well plates coated with matrigel. The cells were imaged under an inverted microscope (Nikon, ECLIPSE Ts2) at 100 magnification and the network length and width was quantified.

### Statistical analyses

All data were expressed as the mean ± S.D. The statistical significance of data was assessed by an unpaired two-tailed t-test. A value of *p* < 0.05 was used as the standard for statistical significance. All experiments were repeated 3 times or more.

## Results

### Ticagrelor and clopidogrel inhibit the expression of inflammatory cytokines induced by LPS

**Fig. 1:**
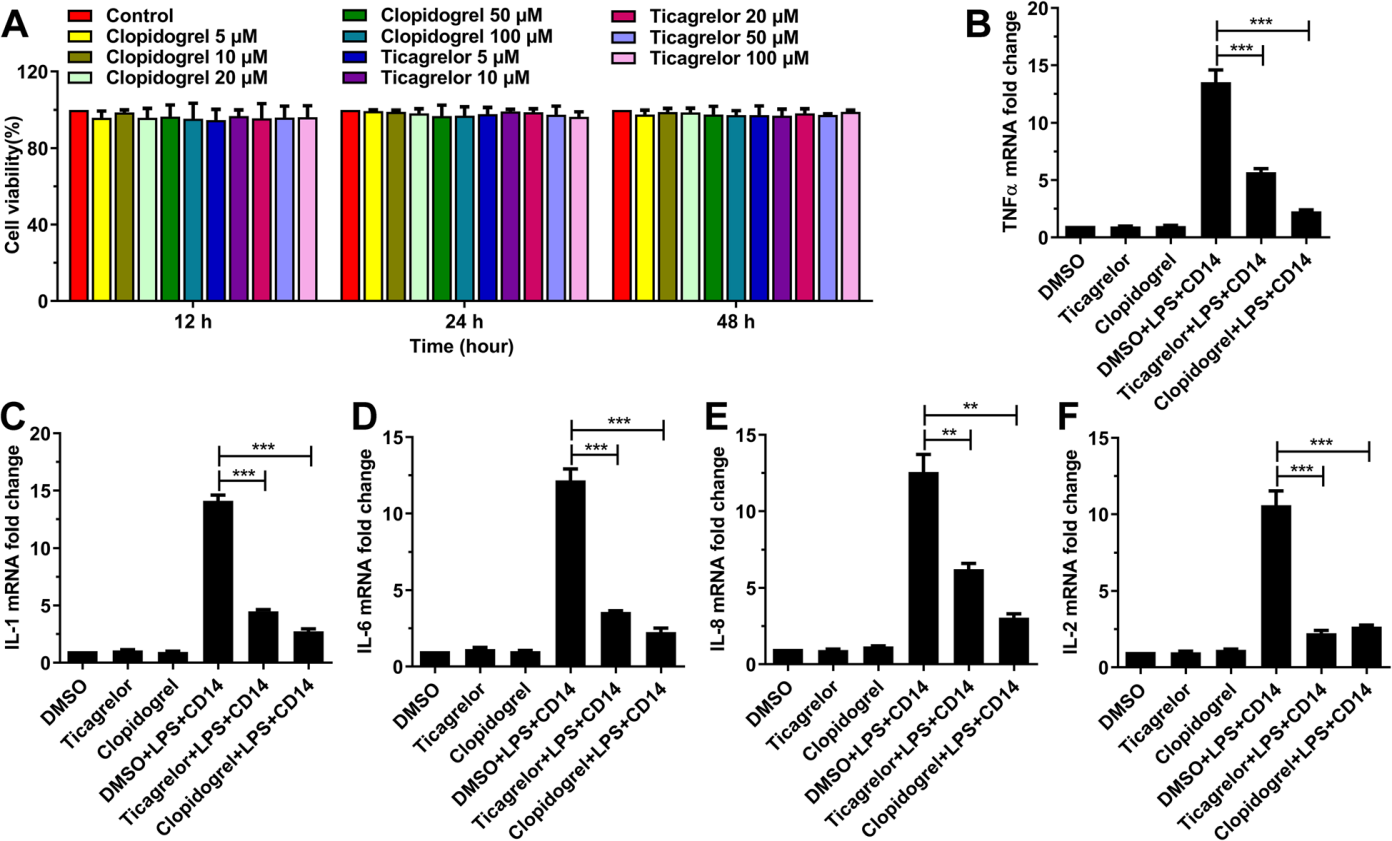
Ticagrelor and clopidogrel inhibit the LPS-induced expression of TNFα, IL-1, IL-6, IL-8, and IL-2. **a**: HUVECs were treated with different concentrations of ticagrelor and clopidogrel, separately, for 12 h, 24 h or 48 h. Cell viability was detected by CCK-8 assays at the indicated time points. **b**-**c**: HUVECs were treated with DMSO, ticagrelor, clopidogrel, DMSO plus LPS and CD14, ticagrelor plus LPS and CD14, and clopidogrel plus LPS and CD14, separately, for 16 h. The mRNA levels of TNFα (**b**), IL-1 (**c**), IL-6 (**d**), IL-8 (**e**), and IL-2 (**f**) were detected by qPCR (*n* = 3; *, *p* < 0.05; **, *p* < 0.01; and ***, *p* < 0.001)

Inflammation plays an important role in the initiation and progression of atherosclerosis, indicating the involvement of inflammatory cytokines in ACS [5]. To test whether ticagrelor and clopidogrel affect the production of inflammatory cytokines, HUVECs were incubated with ticagrelor and clopidogrel at different concentrations (0 μM, 5 μM, 10 μM, 20 μM, 50 μM, 100 μM) for 12, 24 and 48 h separately, without affecting cell viability (Fig. 1a). Then, HUVECs were cultured with different combinations of compounds, DMSO, ticagrelor, clopidogrel, DMSO plus LPS and CD14, ticagrelor plus LPS and CD14, and clopidogrel plus LPS and CD14, separately, for 16 h. The results showed that ticagrelor and clopidogrel inhibited the expression of TNFα, IL-1, IL-6, IL-8, and IL-2 induced by LPS (Fig. 1b-f). These data suggested that ticagrelor and clopidogrel inhibited the production of inflammatory cytokines.

### Ticagrelor and clopidogrel suppress NF-ΚB signaling

**Fig. 2:**
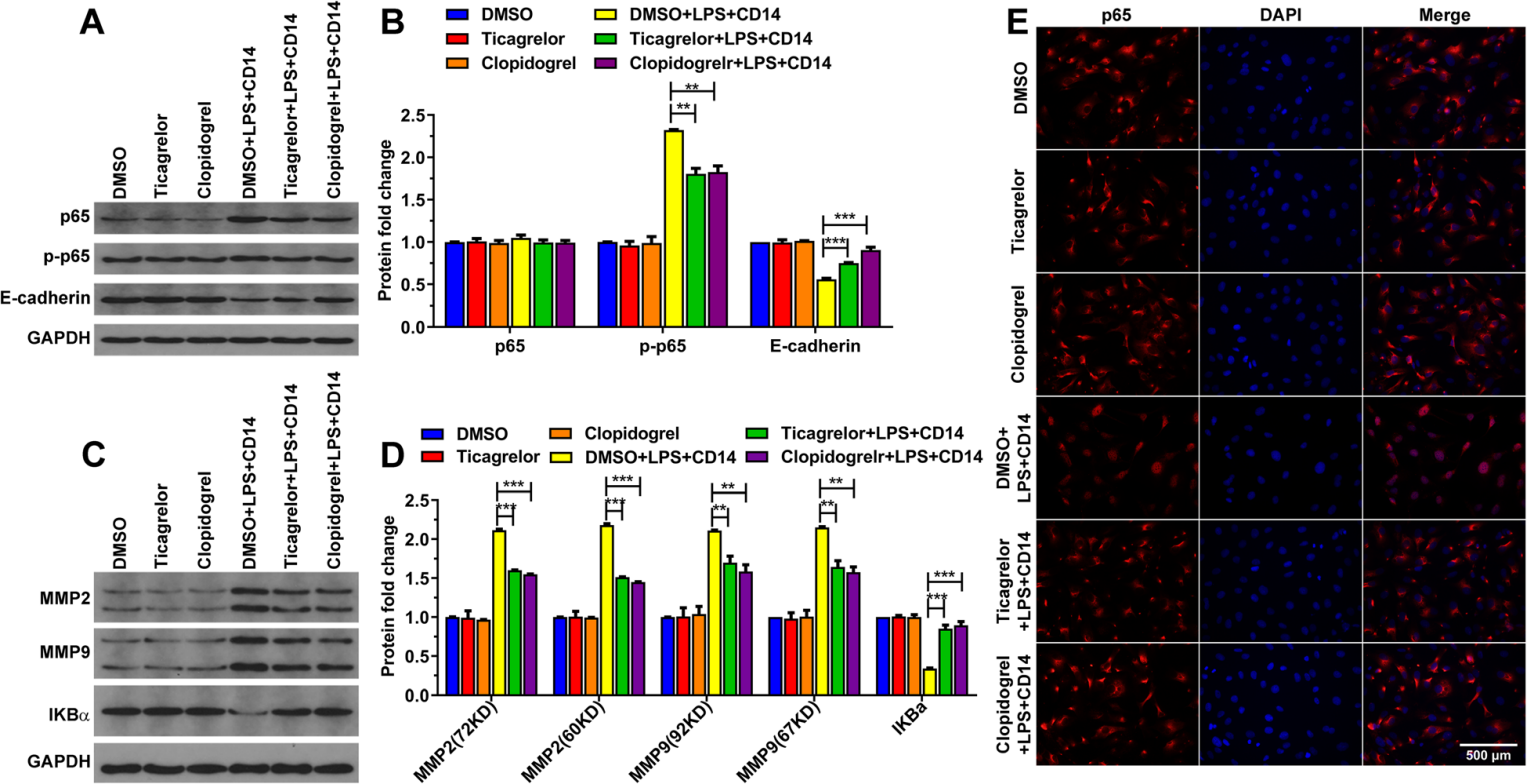
Ticagrelor and clopidogrel inhibit p65 phosphorylation. **a**-**d**: The cells were incubated with the indicated compounds for 16 h, and the cell lysates were analyzed by western blotting. **e**: The cells were incubated with the indicated compounds for 16 h. The localization of p65 in the nuclear and cytoplasmic compartments was detected by fluorescence microscopy. (*n* = 3; ns, not significant, *p* > 0.05; *, *p* < 0.05; **, *p* < 0.01; and ***, *p* < 0.001)

Studies have shown that NF-ΚB is closely related to ACS, and NF-ΚB plays a key role in inflammation [4, 6]. We wondered if ticagrelor and clopidogrel acted on NF-ΚB. The variations of molecules in the NF-ΚB pathway were tested after HUVECs were stimulated with LPS and given ticagrelor or clopidogrel. As a consequence, ticagrelor and clopidogrel inhibited p65 phosphorylation (Fig. 2a-b) and IKBα degradation (Fig. 2c-d). After HUVECs were treated with ticagrelor and clopidogrel, the amount of nuclear-translocated p65 was significantly reduced (Fig. 2e). These findings demonstrated that ticagrelor and clopidogrel inhibited the production of inflammatory factors by suppressing the NF-ΚB pathway.

### Ticagrelor and clopidogrel alleviate cellular dysfunction through suppressing NF-ΚB signaling

**Fig. 3:**
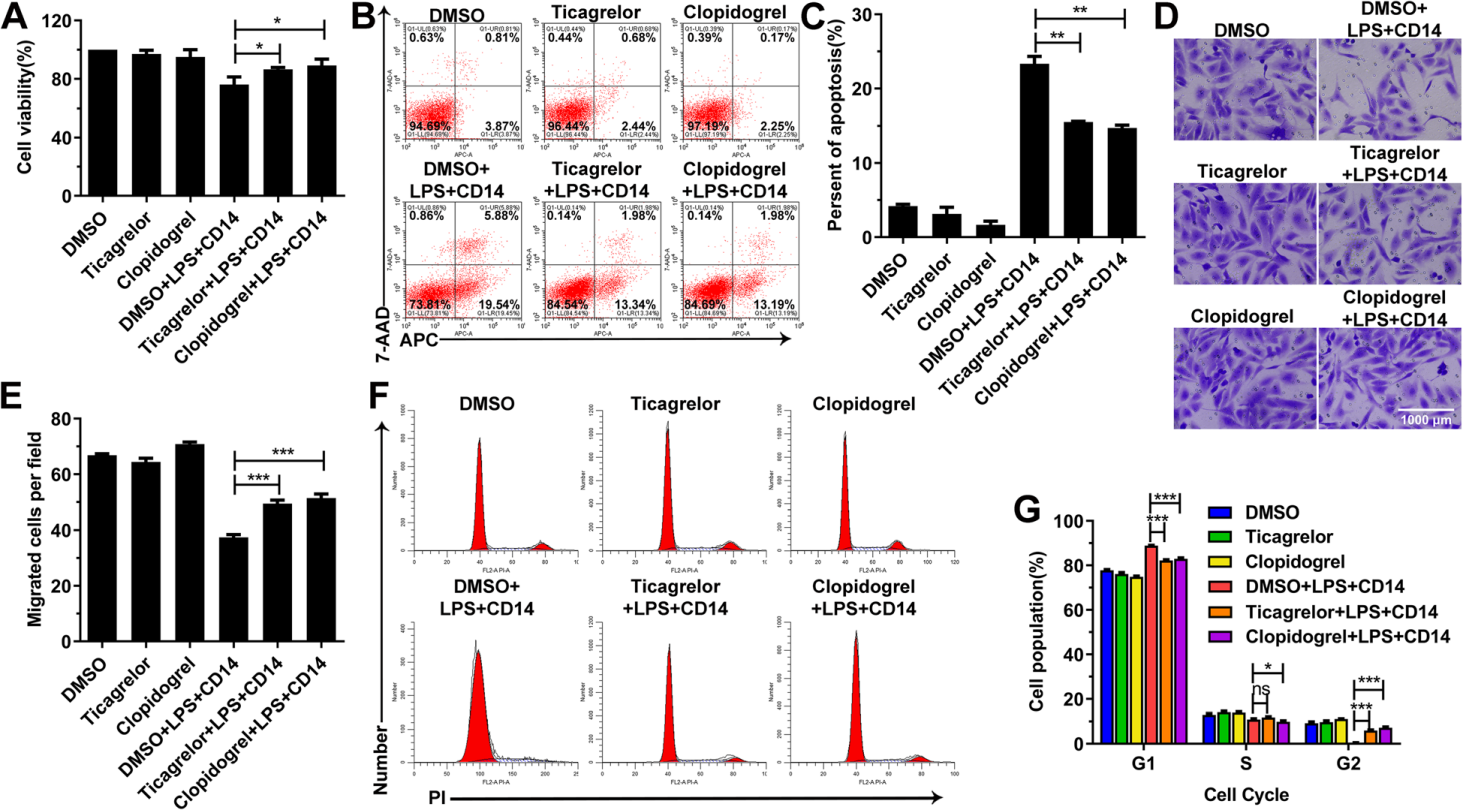
Ticagrelor and clopidogrel inhibit the LPS-induced changes in cell apoptosis, cycle and migration ability. **a**: HUVECs were incubated with the indicated compounds for 16 h, and then, cell viability was determined by CCK-8 assays. **b**-**c**: Flow cytometric analysis of APC/7-AAD staining was used to analyze apoptosis in HUVECs following incubation with the indicated compounds for 16 h. **d**-**e**: After HUVECs were incubated with the indicated compounds for 16 h, cell migration was assessed using a transwell assay, and cells were stained using hexamethylpararosaniline solution. **f**-**g**: HUVECs were incubated with the indicated compounds for 16 h and stained with propidium iodide. DNA content was analyzed by flow cytometry (*n* = 3; ns, not significant, *p* > 0.05; *, *p* < 0.05; **, *p* < 0.01; and ***, *p* < 0.001)

NF-ΚB was shown to be involved in various biological processes, such as cell proliferation, the cell cycle, apoptosis and cell migration, by regulating the expression of various genes [3, 7–9]. Thus, we tested the cell proliferation, cell cycle, apoptosis and cell migration of HUVECs after culture with the indicated compounds (Fig. 3a-g). As shown in Fig. 3a-g, ticagrelor and clopidogrel inhibited apoptosis and the decrease in cell viability caused by LPS (Fig. 3a-c) and restored the cell migration (Fig. 3d-e) and the cell cycle disrupted by LPS (Fig. 3f-g). These data implied that ticagrelor and clopidogrel prevented LPS from damaging cells.

**Fig. 4:**
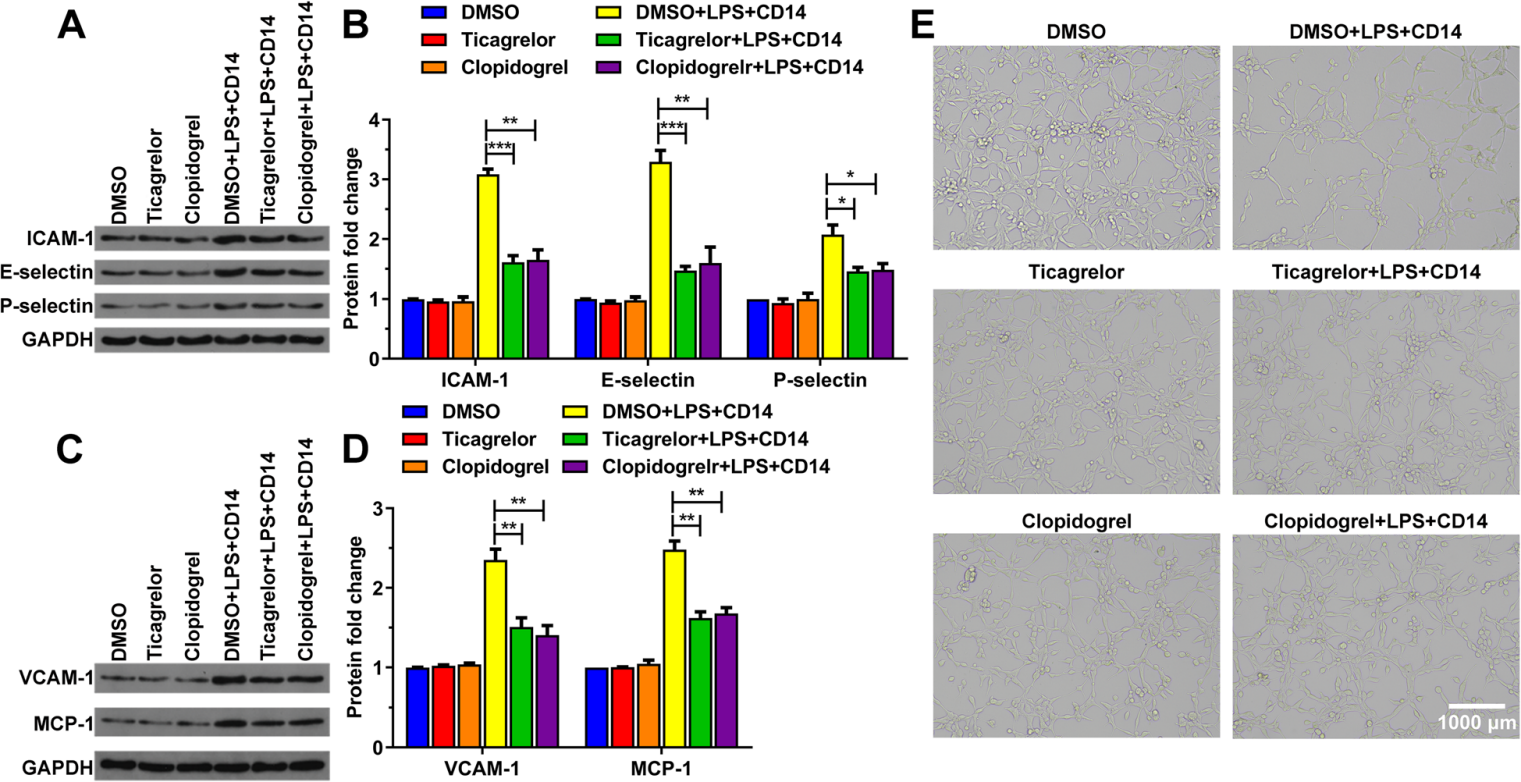
Ticagrelor and clopidogrel restored the LPS-caused reduced ability of HUVECs to form tubular networks. HUVECs were incubated with DMSO (as a control), ticagrelor (20 μM), clopidogrel (20 μM), DMSO plus LPS (10 ng/mL) and CD14 (1 μg/mL), ticagrelor (20 μM) plus LPS (10 ng/mL) and CD14 (1 μg/mL), and clopidogrel (20 μM) plus LPS (10 ng/mL) and CD14 (1 μg/mL), separately, for 16 h. Then, the cell lysates were immunoblotted with the indicated antibodies (**a**-**d**), and tube formation assays were performed using Matrigel as described in the Materials and Methods (**e**). (*n* = 3; ns, not significant, *p* > 0.05; *, *p* < 0.05; **, *p* < 0.01; and ***, *p* < 0.001)

Activated NF-ΚB regulates the expression of a range of genes, including intercellular adhesion molecule-1 (ICAM-1), vascular cell adhesion molecule (VCAM-1), E-selectin, P-selectin, and monocyte chemoattractant protein-1 (MCP-1), which can act directly or indirectly on microvascular endothelial cells or blood cells or mediate their interactions, leading to microcirculation disorders [5, 10]. Therefore, we detected their expression level and the angiogenic capacity of HUVECs treated with DMSO, ticagrelor, clopidogrel, DMSO plus LPS and CD14, ticagrelor plus LPS and CD14, and clopidogrel plus LPS and CD14. We found that ticagrelor and clopidogrel reduced the expression of ICAM-1, E-selectin, P-selectin, VCAM-1 and MCP-1 induced by LPS. (Fig. 4a-d). Furthermore, HUVECs incubated with DMSO, ticagrelor, clopidogrel, ticagrelor plus LPS and CD14, and clopidogrel plus LPS and CD14 formed complex tubular structures, whereas the total width of the tubes was significantly increased in the cells compared with those incubated with DMSO plus LPS and CD14 (Fig. 4g). Our data indicated that ticagrelor and clopidogrel restored the ability of HUVECs to generate blood vessels.

In addition, E-cadherin plays a crucial role in mediating cell adhesion and maintaining the integrity of the vascular endothelial layer. In contrast, MMP2 and MMP9 can degrade the extracellular matrix and basal membrane, damaging the vascular endothelial layer [9, 11, 12]. Hence, we also tested the expression of E-cadherin, MMP2 and MMP9. As shown in Fig. 2a-d, ticagrelor and clopidogrel increased the expression of E-cadherin, which was reduced by LPS and restrained the expression of MMP2 and MMP9 induced by LPS. Ticagrelor and clopidogrel protected the endothelial layer from damage by NF-ΚB.

Overall, our findings reveal that ticagrelor and clopidogrel alleviate cellular dysfunction by suppressing NF-ΚB signaling.

## Discussion

Currently, ticagrelor and clopidogrel are antiplatelet agents and are often used to treat ACS [13, 14]. Ticagrelor reversibly interacts with the P2Y_12_ ADP receptor, which inhibits ADP-induced platelet aggregation by blocking P2Y_12_ receptors, and does not need to be metabolically activated in the liver [15, 16]. Clopidogrel is a prodrug. The metabolite 2-epoxy-clopidogrel is formed by the oxidation of clopidogrel and then forms active metabolites (mercaptan derivatives) through hydrolysis. Clopidogrel selectively inhibits the binding of ADP to its platelet receptor and the subsequent activation of ADP-mediated glycoprotein GPlllb/llla complex, thus inhibiting platelet aggregation [4]. Clopidogrel must undergo biological transformation to inhibit platelet aggregation, but relevant active metabolites have not been isolated. In addition, it was reported that clopidogrel reduces inflammation via inhibition of NF-ΚB activation after severe coronary artery injury in pigs [1]. It can be seen that ticagrelor and clopidogrel can affect other life activities in addition to acting on the P2Y_12_ receptor leading to anticoagulation. We discovered that ticagrelor and clopidogrel protect the endothelial layer by increasing the expression of E-cadherin decreased by LPS and restraining the expression of MMP2 and MMP9 induced by LPS. Thus, ticagrelor and clopidogrel not only inhibit platelet aggregation but also protect endothelial cells. This finding is consistent with the fact that in clinical practice, ticagrelor and clopidogrel are used to prevent thrombosis and improve the nursing care of patients with ACS after PCI.

In recent years, studies have shown that NF-ΚB and VCAM-1 are closely related to the formation of atherosclerosis, causing deviant cell proliferation and inflammatory response, thereby affecting the stability of plaques. Moreover, NF-ΚB was found to be increased in ACS patients. NF-ΚB can transcriptionally regulate key proinflammatory cytokines, chemokines, adhesion molecules, immune recognition receptors and some enzymes in the inflammatory response and directly participate in inflammatory processes, such as inflammatory cell infusion and aggregation [5], injuring vascular endothelial cells [9, 11, 12]. Our study demonstrated that ticagrelor and clopidogrel negatively regulate the NF-ΚB pathway via inhibiting IKBα degradation and p65 phosphorylation, markedly reducing the amount of nuclear-translocated p65, thereby inhibiting cell apoptosis and restoring the cell cycle, cell proliferation, cell migration and formation of blood vessels, protecting cells from the damage by NF-ΚB. Therefore, we speculated that, in addition to inhibiting platelet aggregation, ticagrelor and clopidogrel can reduce the functional impairment of vascular endothelial cells through suppressing NF-ΚB signaling pathway.

Campo et al. found that ticagrelor is superior to clopidogrel in the biological effects of patients with stable coronary artery disease (SCAD) or chronic obstructive pulmonary disease (COPD) [17], while our results suggested that compared with clopidogrel, ticagrelor did not have this advantage in improving LPS-induced endothelial dysfunction. Compared with clopidogrel, there may be another more effective mechanism for ticagrelor relieving cell dysfunction to cure SCAD and COPD. We didn’t have a relevant disease model and cannot directly explain the effects of ticagrelor and clopidogrel inhibiting the NF-ΚB signaling pathway on the treatment of disease. The role of ticagrelor and clopidogrel inhibiting the NF-ΚB signaling pathway in the treatment of disease requires further research. However, the study provided a direction for future research.

## Conclusions

Ticagrelor and clopidogrel negatively regulate the NF-ΚB signaling pathway via inhibiting the degradation of IKBα and the phosphorylation and entry into the nucleus of p65 to alleviate cellular dysfunction, which provides a new theoretical basis for ticagrelor and clopidogrel curing cardiovascular diseases, such as ACS and COPD.

## Data Availability

The datasets generated during and/or analyzed during the current study are available from the corresponding author on reasonable request.

## Abbreviations

ACS: Acute coronary syndrome
ADP: Adenosine diphosphate
HUVECs: Human umbilical vein endothelial cells
LPS: Lipopolysaccharide
PCI: Percutaneous coronary intervention
qPCR: Quantitative polymerase chain reaction
